# Congenital Coronary Artery Anomaly in an Asymptomatic Patient Presenting with Cardiac Arrest

**Published:** 2017-08-30

**Authors:** Taimur Safder, Jared Kvapil, James L. Vacek, Thomas Rosamond

**Affiliations:** 1 University of Kansas School of Medicine-Wichita, Department of Internal Medicine; 2 University of Kansas Medical Center, Department of Internal Medicine, Division of Cardiovascular Diseases

**Keywords:** ALCAPA, Bland White Garland Syndrome, coronary vessel anomalies, heart arrest

## Introduction

Coronary artery anomalies are thought to be present in 0.2% to 1.2% of the general population.^1^ Origin of the left main coronary artery from the pulmonary artery (ALCAPA), also known as Bland-White-Garland syndrome, is a rare coronary artery anomaly (one in 300,000 live births) where the left main coronary artery branches from the pulmonary artery and is unable to supply oxygenated blood to the left side of the heart.^2^,^3^ The clinical presentation of ALCAPA can vary and may present with myocardial ischemia or infarction in children. These ischemic events are thought to be caused by the drop in pulmonary vascular resistance shortly after birth coupled with decreased pulmonary arterial pressure and anterograde flow through the left coronary artery. This can lead to a “coronary steal” phenomenon where collateral flow from the right coronary artery fills the left main system but the myocardium, overall, remains inadequately perfused. These patients, if left untreated, can have a mortality of up to 90% in the first year of life.

While there are very little data about long term outcomes, early identification and surgical correction is believed to lead to a good prognosis and myocardial tissue recovery.^4^ While ALCAPA is mainly a pediatric disease, approximately 10 – 15% of all cases can present in adults where survival is determined by the dominance of the right coronary artery (RCA) and the level of inter-coronary collaterals. There is an estimated sudden death rate among these cases of 80 – 90% at the mean age of 35.^4^,^5^

## Case Report

A 48-year-old asymptomatic Caucasian female with no prior significant medical history was transferred to the University of Kansas Medical Center in critical condition. The patient collapsed after dancing in a club and cardiopulmonary resuscitation was performed for approximately 6 – 8 minutes. The presenting rhythm was ventricular fibrillation and two rounds of electrical cardioversion were administered after which return of circulation was achieved. The patient was transported by emergency medical services and reported to be stable during transport, but was in ventricular fibrillation at the time of hospital arrival. Two more rounds of electrical cardioversion were administered successfully as the patient returned to sinus rhythm.

At arrival to the hospital, the patient was intubated and therapeutic hypothermia treatment was initiated. Urine drug screen was positive for cannabinoids and alcohol level was less than 10 mg/dL. Brain natriuretic peptide (BNP) was elevated to 859 pg/ml; initial troponins were 0.12 (normal < 0.05). A 2-dimensional (2D) Doppler echocardiogram demonstrated severely depressed systolic function and global hypokinesis with an estimated 15% ejection fraction (EF).

**Figure 1:**
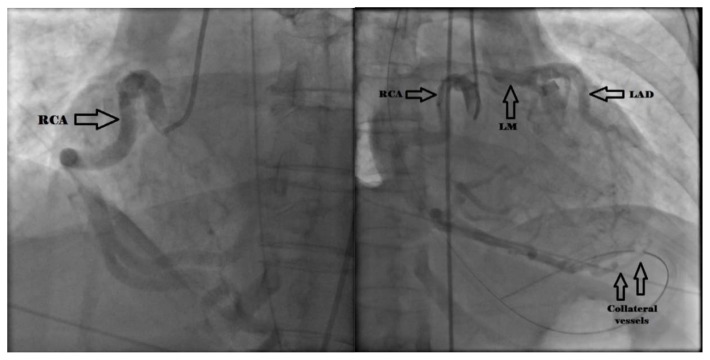
Sequential images from coronary angiography demonstrated contrast injection (left) into a dilated right coronary artery (RCA) with flow via (right) collateral vessels into the left anterior descending (LAD) and left main coronary arteries (LM).

**Figure 2:**
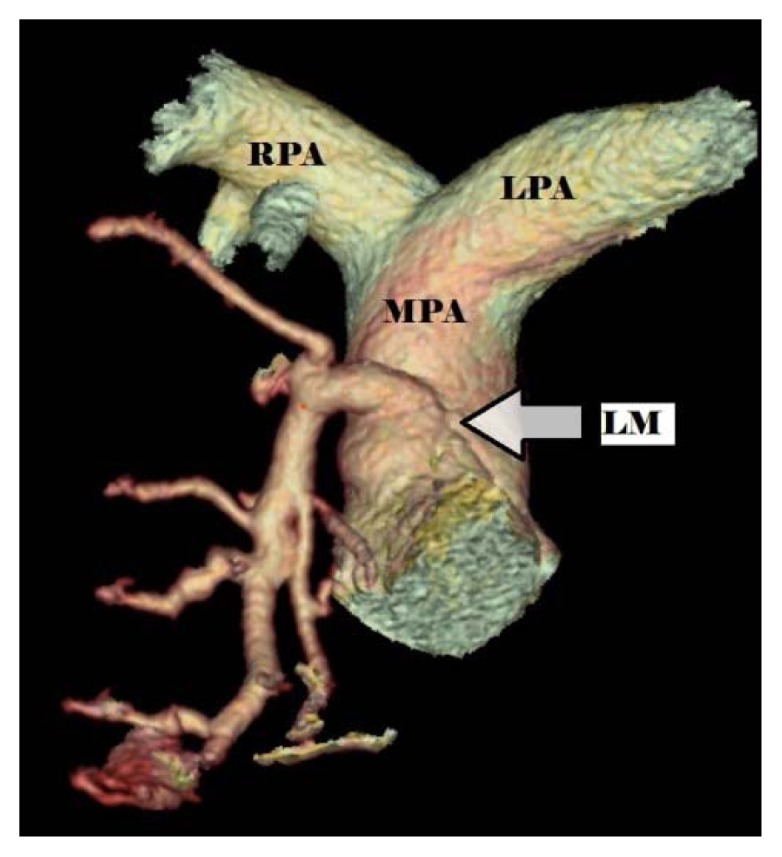
CT reconstruction illustrated left main coronary artery (LM) originating from main pulmonary artery (MPA). Right pulmonary artery (RPA) and left pulmonary artery (LPA) branched off the MPA.

Computed tomography (CT) imaging of the head and spine showed no abnormalities while an electrocardiogram showed a 2 mm ST depression in the lateral leads without any significant T wave changes. The patient was taken urgently for heart catheterization. No significant atherosclerotic lesions were found, but she had a large right coronary artery with collaterals filling the left coronary system (Figure 1). A CT coronary angiogram demonstrated the origination of the left main coronary artery from the main pulmonary artery (Figure 2). A dominant right coronary artery was noted again providing collaterals to the left coronary circulation. A diagnosis of anomalous left coronary artery from the pulmonary artery (ALCAPA) was established.

**Figure 3:**
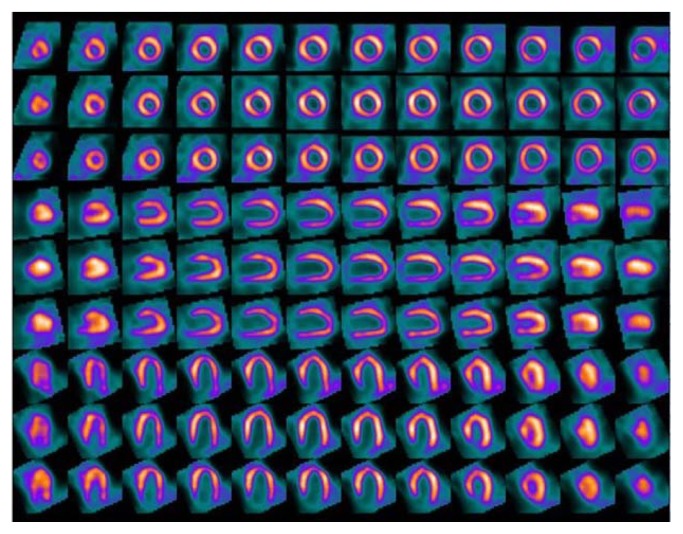
Resting thallium study (24 hours post injection) demonstrated no marked perfusion defects.

The case was discussed with the cardiothoracic surgery team who recommended a viability study before evaluation for surgery for possible revascularization. Accordingly, a resting and delayed myocardium viability study was ordered two days after initial cardiac arrest to guide surgical treatment options. Surprisingly, the stress thallium study demonstrated normal viability in all segments (Figure 3). To corroborate the viability study findings, a follow-up 2D Doppler echocardiogram three days after cardiac arrest showed normal systolic function and a recovered EF at 50%. The patient was extubated on day two of hospitalization and had no notable neurologic sequela from cardiac event.

The patient’s in-hospital recovery was unremarkable other than a few episodes of non-sustained ventricular tachycardia on day four of hospitalization. There were several issues complicating a surgical intervention in our patient as she was from out of state and in town for a family visit, uninsured, and preferred to go back home for any major surgical procedure. After extensive discussions between the multi-disciplinary medical team and the patient, it was decided that the patient would receive her surgical intervention in her home state and an appointment was made with a cardiothoracic surgeon there. A single chamber implantable cardioverter defibrillator (ICD) device was placed for secondary prevention of sudden death from cardiac arrest before discharge on day six of hospitalization.

## Discussion

ALCAPA in adults presents with a varying degree of symptoms such as dyspnea at rest or with exertion, syncope, cardiac palpitations or arrhythmias, and angina pectoris.^6^ Our patient denied experiencing any of those symptoms prior to her sudden cardiac arrest episode owing most likely to sufficient blood flow via collateral vessels via the enlarged RCA (Figure 1). Only a handful of cases have been reported where the presenting symptom for a patient with ALCAPA has been a ventricular arrhythmia precipitated by activity, as is the case for our patient.^7^,^10^

Two mechanisms have been proposed as the cause of these arrhythmias.^7^ First, hypo-perfusion due to the coronary anomaly could cause a small myocardial infarction which would lead to scar formation and alteration of the conduction system. Second, an arrhythmia could be triggered by an acute ischemic episode during exercise or strenuous physical activity. The latter most likely would explain our patient’s presentation. The transient ischemic event experienced by our patient most likely was induced by her increased level of activity. This sudden increase in activity seems to be atypical for our patient as she was overweight and reported a very sedentary lifestyle for most of her adult life. These cases seem to indicate the presence of sufficient collaterals is a deterrent for symptoms, but does not seem to protect against sudden cardiac arrest due to ventricular arrhythmias.^7^

Surgical correction of the anomaly soon after diagnosis is considered optimal treatment and most techniques aim to establish a two coronary system.^7^,^8^ Mitral regurgitation is a common consequence of ALCAPA and thought to result from ischemic damage to papillary muscles, but there is insufficient evidence if simultaneous mitral valve replacement with surgical anomaly correction leads to better outcomes.^11^ Some cases have been reported where surgical correction of the anomaly was not undertaken and medical management was considered more appropriate due to the patient’s age, comorbidities, and level of collateralizations among other factors.^6^,^9^ While our patient did not fall in that group, due to circumstances surrounding the case and personal preferences as discussed, the patient was discharged with an ICD device and would follow-up in her home state for a surgical intervention to establish a two coronary system.

In summary, ALCAPA is a rare subset of coronary anomalies and awareness of it can be essential when dealing with arrhythmias in the absence of atherosclerotic heart disease. Early diagnosis and treatment can yield promising results. Our patient had been asymptomatic prior to her episode of sudden cardiac arrest, likely due to sufficient blood flow from her enlarged right coronary collateral vessels. Surgical correction of the anomaly soon after diagnosis is considered optimal treatment and most techniques aim to establish a two coronary system. Our case presented not only a rare condition in ALCAPA but an uncommon presentation with an asymptomatic adult patient with a sudden cardiac arrest.
